# Effectiveness and safety of carbohydrate counting in the management of adult patients with type 1 diabetes mellitus: a systematic review and meta-analysis

**DOI:** 10.20945/2359-3997000000045

**Published:** 2018-05-07

**Authors:** Eliege Carolina Vaz, Gustavo José Martiniano Porfírio, Hélio Rubens de Carvalho Nunes, Vania dos Santos Nunes-Nogueira

**Affiliations:** 1 Universidade Estadual Paulista Universidade Estadual de São Paulo Faculdade de Medicina de Botucatu Departamento de Clínica Médica Botucatu SP Brasil Departamento de Clínica Médica, Faculdade de Medicina de Botucatu, Universidade Estadual de São Paulo (Unesp), Botucatu, SP, Brasil; 2 Universidade Federal de São Paulo Universidade Federal de São Paulo Centro Cochrane do Brasil, Disciplina de Medicina de Urgência e Medicina Baseada em Evidências São Paulo SP Brasil Centro Cochrane do Brasil, Disciplina de Medicina de Urgência e Medicina Baseada em Evidências, Universidade Federal de São Paulo (Unifesp), São Paulo, SP, Brasil; 3 Universidade Estadual Paulista Universidade Estadual de São Paulo Faculdade de Medicina de Botucatu Departamento de Saúde Pública Botucatu SP Brasil Departamento de Saúde Pública, Faculdade de Medicina de Botucatu, Universidade Estadual de São Paulo (Unesp), Botucatu, SP, Brasil

**Keywords:** Type 1 diabetes mellitus, carbohydrate counting, quality of life, systematic review, meta-analysis

## Abstract

**Objective:**

This study aimed to evaluate the effectiveness and safety of carbohydrate counting (CHOC) in the treatment of adult patients with type 1 diabetes mellitus (DM1).

**Materials and methods:**

We performed a systematic review of randomized studies that compared CHOC with general dietary advice in adult patients with DM1. The primary outcomes were changes in glycated hemoglobin (HbA1c), quality of life, and episodes of severe hypoglycemia. We searched the following electronic databases: Embase, PubMed, Lilacs, and the Cochrane Central Register of Controlled Trials. The quality of evidence was analyzed using the Grading of Recommendations Assessment, Development and Evaluation (GRADE).

**Results:**

A total of 3,190 articles were identified, and two reviewers independently screened the titles and abstracts. From the 15 potentially eligible studies, five were included, and 10 were excluded because of the lack of randomization or different control/intervention groups. Meta-analysis showed that the final HbA1c was significantly lower in the CHOC group than in the control group (mean difference, random, 95% CI: −0.49 (-0.85, −0.13), p = 0.006). The meta-analysis of severe hypoglycemia and quality of life did not show any significant differences between the groups. According to the GRADE, the quality of evidence for severe hypoglycemia, quality of life, and change in HbA1c was low, very low, and moderate, respectively.

**Conclusion:**

The meta-analysis showed evidence favoring the use of CHOC in the management of DM1. However, this benefit was limited to final HbA1c, which was significantly lower in the CHOC than in the control group.

## INTRODUCTION

Diabetes mellitus (DM) comprises a heterogeneous group of metabolic disorders that commonly feature hyperglycemia, which results from disturbances in insulin secretion, insulin action, or both (1). In most cases, type 1 DM (DM1) is an autoimmune disease characterized by the destruction of insulin-producing beta cells, accounting for 5% to 10% of all DM cases (1). In Brazil, eight of every 100,000 people under the age of 20 have DM1 (2).

The therapeutic treatment and control of DM1 includes the use of insulin for glycemic control, balanced diet, and regular physical activity. Daily insulin requirements vary based on age, diet, patient self-monitoring of blood glucose and daily routines.

Glycemic control of patients with DM is important because it impacts the development of diabetic complications (3). Diabetes control is evaluated mainly according to the levels of HbA1c, fasting blood glucose, and postprandial blood glucose (blood glucose measured two hours after meal consumption). Borderline normal values without the risk of hypoglycemia, impaired mental status, and patient welfare indicate good glycemic control (4).

The American Diabetes Association recommends the following levels for nonpregnant adults: HbA1c < 7%, preprandial capillary plasma glucose between 80 mg/dL and 130 mg/dL, and peak postprandial capillary plasma glucose < 180 mg/dL (4). The Diabetes Control and Complications Trial (DCCT) showed that adequate glycemic control in patients with DM1 (e.g., fasting blood glucose levels up to 110 mg/dL, postprandial glucose levels lower than 180 mg/dL, and HbA1c < 6.5%) delays the onset and progression of microvascular complications, such as retinopathy, nephropathy, and neuropathy, and reduces the risk of any cardiovascular event by 42% and that of nonfatal infarction, stroke, and death by 57% (3).

The treatment of patients with DM1 facilitates proper development in children and adolescents and improves the quality of life (QOL) of patients in general (5).

DM1 control cannot be achieved solely via regular insulin use. Combining insulin use with diet and physical activity is important. In particular, adjusting insulin therapy to an individualized food plan is key to proper metabolic control (3). Conventional nutritional advice for patients with DM1 is the same as for the general population. Specifically, a balanced nutrition with appropriate concentrations of macro- and micronutrients should be based on the goals of treatment (i.e., total carbohydrate (CHO), 45%-60% of total energy intake (VET); protein, 15%-20% of VET; total fat (GT), up to 30% of VET; and minimum dietary fiber, 20 g/day or 14 g/1000 kcal) (6).

In addition to conventional nutritional DM1 treatments, carbohydrate counting (CHOC) is a meal planning tool that allows for great variation in food choices among individuals with DM (7), with the main objective of providing flexibility in food intake (8). Few dietary restrictions and the option to decide the number of meals (traditional treatment plans recommend eating six meals per day) may improve acceptance of the disease and overall QOL (9).

CHOC consists of measuring the amount of carbohydrates to be eaten during every meal in grams. Based on that count and preprandial blood glucose levels, the patient calculates the dose of fast or regular insulin they need before each meal (10,11). This method can be used for any patient with diabetes in combination with the use of varying doses of rapid-acting insulin or continuous subcutaneous insulin infusion (12). Two CHOC methods are widely used: listing carbohydrate equivalents (A) and measuring the carbohydrate in grams (B). In method A, foods are grouped so that each food portion chosen by the patient corresponds to 15 g of carbohydrate, classifying them as equivalents. Method B consists of the sum of carbohydrate grams in each food per meal based on information in food labels and tables (13).

To improve glycemic control and decrease the frequency of acute and chronic complications, CHOC is now recommended as another nutritional tool (3,14).

Regarding the efficacy of the CHOC method in metabolic DM1 control in the DCCT study, individuals who adjusted their pre-meal insulin doses based on carbohydrate counts had a 0.5% decrease in HbA1c compared to the group that used a fixed dose (15). Dias and cols. (16) showed that HbA1c levels were reduced in a group of 55 adult patients, and although the total daily dose of insulin increased, no weight gain was observed. Waller and cols. (17) also evaluated CHOC in children and adolescents with DM1 and reported no changes in HbA1c, body mass index (BMI), or frequency of hypoglycemic episodes. However, the children and their parents showed an improvement in QOL.

We hypothesized that the CHOC method in adult individuals with DM1 may be more effective and efficient for glycemic control and better improve QOL compared to conventional nutritional guidance.

This study aimed to evaluate the effectiveness and safety of CHOC in the treatment of adult patients with DMI using a systematic literature review.

## MATERIALS AND METHODS

This review was performed according to Cochrane Methodology (18) and reported according to the PRISMA Statement (19).

### Eligibility criteria

We included randomized controlled trials with at least three months of follow-up, and evaluation of outcomes in which patients were randomly divided into two groups, intervention or comparison. Data were interpreted based on patient-characteristics, intervention, comparison, and outcomes (PICO) as described below.

### Patients

Patients consisted of men and women aged over 18 years old who had been diagnosed with DM1 for at least six months and were not in the “honeymoon period”, in which the pancreas can produce small amounts of insulin that can be enough to achieve adequate glycemic control at a daily dose of less than 0.5 IU insulin/kg in 24 hours. Patients had standard nutritional counseling with a professional nutritionist and took slow-acting or intermediate and multiple fast or regular insulin doses before meals (breakfast, lunch, and dinner) or continuous subcutaneous insulin infusion (CSII). Studies that included pregnant women, individuals with a BMI > 40 kg/m², kidney failure, or HbA1c >14% were excluded from analysis.

### Intervention

Individuals in the intervention group had nutritional counseling for CHOC to determine the amount of fast or regular insulin that they would need before each main meal.

### Comparison

The comparison group included individuals who had conventional nutritional advice and used fixed doses of fast or regular insulin before meals.

### Outcomes

Assessed outcomes were reduction in HbA1c, frequency of severe hypoglycemia, improved QOL, body weight or BMI gain, lipid profile, and total daily dose of insulin. Validated questionnaires were used to evaluate QOL: Audit of Diabetes-Dependent Quality of Life (ADDQoL), Diabetes Treatment Satisfaction Questionnaire (DTSQ), and Diabetes Quality of Life Measure (DQoL).

### Search strategy and selection

No language restriction was imposed. We searched the following electronic databases through November 30, 2016 to identify randomized clinical trials involving CHOC versus conventional nutritional advice in the treatment of DM1 patients: Embase (1980-2016), PubMed (1966-2016), Lilacs (1982-2016), and the Cochrane Central Register of Controlled Trials (CENTRAL, the Cochrane Library, issue 2016). We also searched for ongoing clinical trials on the clinicaltrials.gov website. Medical Subject Heading terms used included “Type 1 Diabetes Mellitus”, “Carbohydrates”, “Nutrition Therapy”, and “Randomized Controlled Trial”.

Two reviewers (ECV and VSNN) independently screened the titles and abstracts identified in the literature search. Studies potentially eligible for inclusion in the review were selected for complete reading.

### Data extraction and risk of bias

Both reviewers assessed the study quality and extracted data using an extraction template. For each trial, we assigned the risk of bias considering the quality scores for random sequence generation, allocation concealment, blinding of outcome assessment, and incomplete outcome data. We used the criteria described in the Cochrane Reviewer's Handbook (18) to classify these scores as adequate (low risk of bias), unclear, and inadequate (high risk of bias).

### Data synthesis and analysis

We performed the meta-analysis by using a random-effects model in Review Manager 5.3 software. For dichotomous outcomes, the relative risk was calculated with a 95% confidence interval and continuous variables were expressed as a weighted mean difference with 95% confidence intervals. Potential causes of heterogeneity among studies were also analyzed. The I^2^ statistic was used to measure the impact of heterogeneity for each outcome (where an I^2^ ≥ 50 indicates a considerable level of heterogeneity) (18). When we found heterogeneity, we attempted to determine possible reasons for it via subgroup analysis or by examining individual studies.

### Quality assessment

The quality of evidence per outcome measurement was graded according to the Grading of Recommendations Assessment, Development and Evaluation (GRADE) Working Group. The confidence of the GRADE system decreases if randomized studies have major limitations that may interfere with treatment effect estimates (20). These limitations include risk of bias for each study, inconsistency, indirectness, imprecision, and publication bias of each evaluated outcome per GRADE considerations.

## RESULTS

**Figure 1:**
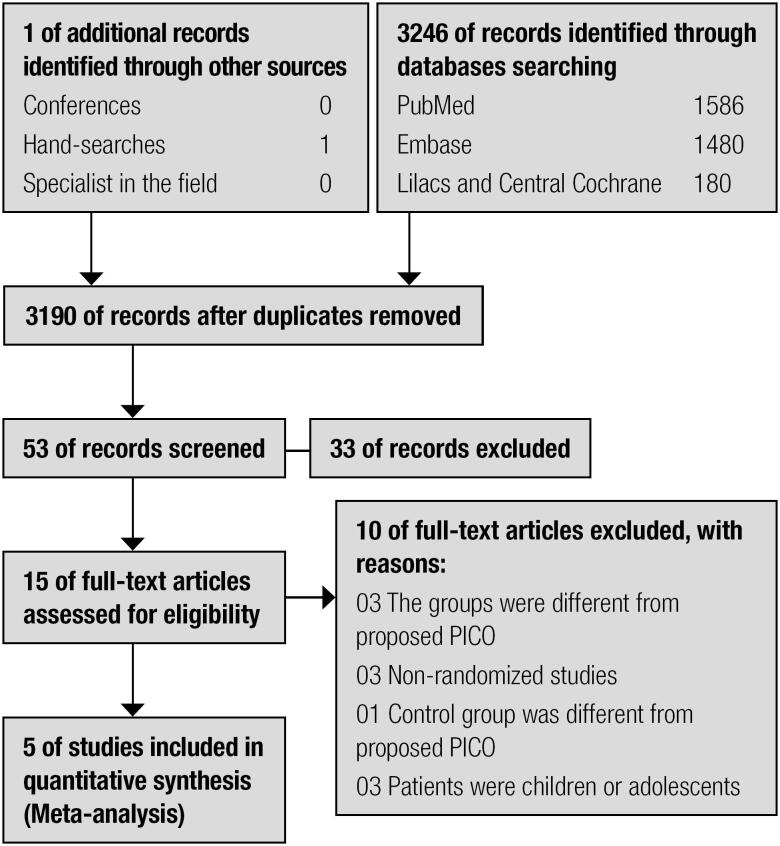
Flowchart for identifying eligible studies

From the database searches, 3190 articles were identified (Figure 1). Fifteen articles were potentially eligible for inclusion in the analysis and were selected for full review. Five of the 15 studies were included for analysis (7,10,15,21,22). Of the 10 excluded studies, three were not randomized (23-25), three compared two different methods for mealtime insulin dosing; no group had conventional nutritional advice using a fixed dose of fast or regular insulin before meals (8,26,27). In three studies, patients were children or adolescents (28-30), and one study compared three different possibilities of insulin self-adjustments, without a group using a fixed dose of fast or regular insulin before meals (31).

The baseline characteristics of study participants and eligibility criteria of the included studies are presented in Tables 1 and 2, respectively. P values < 0.05 were considered statistically significant.

**Table 1:** Baseline characteristics of patients in each included study

Study	Number of randomized patients	Male/Female	Age (SD)	HbA1C (SD)	Fasting Glucose nmol/L (SD)	BMI or weight (kg) (SD)	Insulin dose (SD)
Dafne, 2002	G1 = 84	-		G1- 9.4 (1.2)	-	G1 = 80.5 (1.7)	-
	G2 = 85		-	G2 = 9.3 (1.1)		G2 = 77.4 (13.4)	
Laurenzi, 2011	G1 = 28	G1 = 15/13	G1 = 41.2 (10.0)	G1 = 7.9 (0.9)	-	G1 = 23.7 (21-25.2)	**G1 = 36 (24.5-49)
	G2 = 28	G2 = 09/19	G2 = 39.8 (9.8)	G2 = 8.1 (1.5)		G2 = 23.8 (20.8-26.8)	**G2 = 33 (28.5-39.5)
Scavone, 2010	G1 = 100	G1 = 49/51	G1 = 39 (11)	G1 = 7.8 (1.3)	-	-	-
	G2 = 156	G2 = 74/82	G2 = 39 (11)	G2 = 7.5 (0.8)			
Schmidt, 2012	G1 = 26	G1 = 10/11	G1= 41 (10)	G1 = 9.2 (0.6)	-	-	*G1 = 0.6 (0.2)
	G2 = 09	G2 = 06/02	G2 = 46 (09)	G2 = 9.1 (0.7)			*G2 = 0.7 (0.17)
Trento, 2009	G1 = 27	G1= 18/9	G1 = 37.33 (12.6)	G1 = 7.6 (1.3)	G1 = 9.64 (5.17)	G1 = 24.4 (2.6)	**G1 = 47.9 (10.6)
	G2 = 29	G2 = 12/17	G2 = 36.76 (7.9)	G2 = 7.7 (1.24)	G2 = 9.05 (5.08)	G2 = 23.5 (3.3)	**G2 = 45.7 (12.6)

G1: intervention group; G2: control group.

* Daily insulin dose per kg;

** Total insulin dose (basal and bolus).

- No information provided.

**Table 2:** Length of follow-up, inclusion criteria, and outcomes of included studies

Study	Follow-up	Inclusion criteria	Outcomes
DAFNE, 2002	6 months	> 18 years of HbA1c from 7.5% to 12% and diagnosis greater than 2 years without advanced complications	Change in HbA1C (HPLC), severe hypoglycemia and hyperglycemia, quality of life (ADDQoL, DTSQ, and W-BQ12), weight, blood pressure, lipid profile, injections, glucose monitoring, and daily total dose of insulin
Laurenzi, 2011	3 and 6 months	Age between 18 and 65 years and treatment with continuous insulin infusion pump for more than 3 months	Change in HbA1C (HPLC), hypoglycemia, quality of life (DSQOLS), BMI, waist, fasting glucose, and daily insulin dose
Scavone, 2010	9 months	Diagnosis of type 1 diabetes mellitus over 5 years	Changes in HbA1c, hypoglycemia, daily insulin dose, weight, lipid profile, creatinine, and microalbuminuria
Schmidt, 2012	4 months	Age between 18 and 65 years, poor metabolic control, diabetes duration over 12 months, and use of basal and fast analogue insulin.	Changes in HbA1C, severe hypoglycemia, treatment satisfaction and perceived frequency of hypo- and hyperglycemia (DTSQs and DTSQc), quality of life (ADDQoL), change in the perception of problem areas (PAID), and change in fear of hypoglycemia (HFS)
Trento, 2009	30 months	Age <70 years, onset of diabetes before 30 years of age, and onset of insulin use within the first year of the diagnosis	Changes in HbA1C (HPLC), severe hypoglycemia and hyperglycemia, quality of life (DQOL, GISED, CSI), BMI, and lipid profile and fasting glucose

Dafne and cols. (7) performed a single-center study in England. A total of 169 patients with DM1 who had been diagnosed more than two years prior without chronic complications and intensive insulin therapy were randomized to CHOC or conventional nutritional treatment. The main outcome measures after a six-month follow-up were: HbA1c, severe hypoglycemia, and the impact of diabetes on QOL as assessed using the ADDQoL questionnaire.

Laurenzi and cols. (10) recruited patients from a clinic in Milan, Italy. A total of 61 adult patients with DM1 who had been treated with CSII were randomly assigned to learn CHOC in the intervention group or to estimate pre-meal insulin doses empirically for six months. The main outcome measures were: HbA1c, fasting glucose, BMI, waist circumference, daily insulin dose, hypoglycemic events, and analysis of QOL through the Diabetes-Specific Quality-of-life Scale, which evaluates individual treatment goals in patients with DM1.

In the study of Scavone and cols. (Italy) (21), 256 patients with DM1 who had been diagnosed for more than five years were randomized to a CHOC group or a control group. Weight, BMI, HbA1c, lipid profile, uric acid, creatinine, microalbuminuria, daily insulin requirements, and number of episodes of hypoglycemia (blood glucose < 70 mg/dL) were the main outcomes evaluated.

Schmidt and cols. (15) recruited patients from two centers in Denmark. The authors randomized 63 adults with DM1 and poor metabolic control (HbA1c: 8.0% to 10.5%) to the CHOC or control groups for more than 12 months using analogues of basal and fast insulin. The main outcome measures were: change in HbA1c, weight, satisfaction with the treatment of diabetes, and perceived frequency of hyper- and hypoglycemia. The parameters were measured according to the Diabetes Treatment Satisfaction status version and version change questionnaires (DTSQs and DTSQc, respectively). QOL was analyzed using the ADDQoL questionnaire.

Trento and cols. (22) included 56 patients with DM1 who had all been diagnosed before age 30 years. Twenty-seven subjects were randomized to a CHOC program and the remaining patients were assigned to the control group. Body weight, fasting glucose, HbA1c, total cholesterol, high-density lipoprotein cholesterol, triglycerides and creatinine, frequency of hypoglycemia, and QOL were the main outcome measures.

### Risk of bias

Dafne and cols. (7) and Laurenzi and cols. (10) randomized patients using a computer-generated random number. Schmidt and cols. (15) performed the random distribution with a 1: 3: 3 ratio in blocks of 14 with sealed, opaque envelopes containing group assignments. Scavone and cols. (21) and Trento and cols. (22) did not describe how the randomization sequence was generated.

Only Dafne, Laurenzi, and Schmidt described allocation concealment and as such were classified as low risk of bias. The other two studies did not provide any information regarding the allocation process.

Most of the studies included did not report blinding for outcome evaluation. However, except for severe hypoglycemia, most were laboratory assessments, which were not susceptible to bias. QOL questionnaires were self-applied and could not be blinded.

Only Laurenzi and cols. reported that patients who did not complete the treatment regimen were included in the final analysis (low risk) (10). Trento and cols. (22) reported that all participants completed the treatment (low risk). In the study of Dafne and cols. (7), 28 patients were lost to follow-up and were not included in the final analysis, although the number of patients who were lost was not significantly different between the groups (15 in the intervention group and 13 in the control group) (low risk). Scavone and cols. (21) had a 27% loss of patients in the intervention group, and they were not included in the final analysis (high risk). Schmidt and cols. (15) had a 19% loss, and these patients were not included in the final analysis (high risk).

### Meta-analysis of outcomes

**Figure 2:**
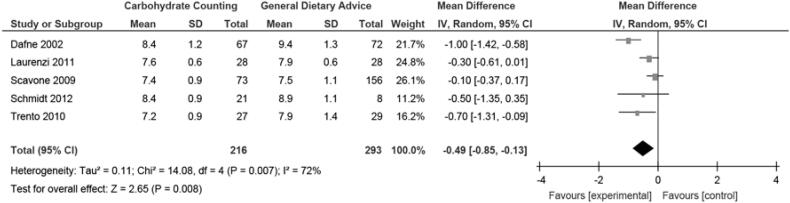
Meta-analysis of change in HbA1c.

The five studies included analyzed changes in HbA1c levels at the end of the study. Meta-analysis showed that the final HbA1c was significantly lower in the CHOC group than in the control group (mean difference, random, 95% CI: −0.49 (-0.85, −0.13), p = 0.006, I^2^ = 72%) (Figure 2).

**Figure 3:**
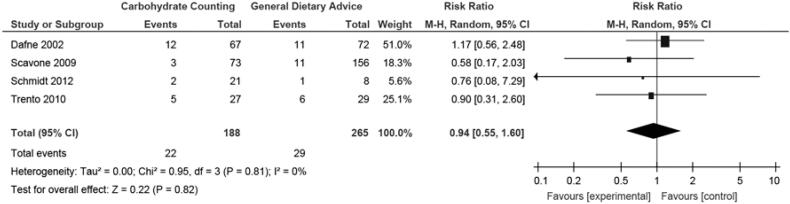
Meta-analysis of episodes of severe hypoglycemia.

In the four trials, the number of patients who experienced at least one episode of severe hypoglycemia can be assessed (7,15,21,22). The meta-analysis of this outcome was not significantly different between groups (risk ratio, random, 95% CI: 0.94 (0.55, 1.6), p = 0.82, I^2^ 0%) (Figure 3).

Regarding QOL, two studies (7,15) used the ADDQoL instrument, but no difference was noted between groups (mean difference, random, 95% CI: −0.23 (-1.4, 0.94), p = 0.7, I^2^ = 84%). The same studies also used the DTSQs questionnaire and found no difference between groups (mean difference, random, 95% CI: 3.53 (-7.11, 14.16), p = 0.52, I^2^ = 95%).

We plotted the QOL outcomes using different questionnaires (DQOL and DTSQs) cited in three studies (7,15,22,23) and found no significant differences between groups (std. mean difference, random, 95% CI: 0.64 (-0.7, 1.98), p = 0.35, I^2^ = 94%).

Meta-analysis of total cholesterol, HDL-C, and triglycerides could only be performed based on the results reported in the study of Dafne and cols. (7) and Trento and cols. (22); no significant differences were noted among groups.

According to the GRADE, the quality of evidence of the primary outcomes was moderate for changes in HbA1c, low for episodes of severe hypoglycemia, and very low for QOL (Table 3).

**Table 3:** Quality of evidence of primary outcomes according to GRADE approach

Outcomes	Risk of bias	Inconsistency	Indirectness	Imprecision	Publication bias	Intervention vs comparator 95% CI	Participants (studies)	Quality of evidence
Severe hypoglycemia	Serious* (-1)	No	No	Serious (-1)***	Unlikely	RR 0.92 (0.54 a 1.56)	453 (4)	+Low
Quality of Life (ADDQoL)	Serious* (-1)	Serious (-1)**	No	Serious (-1)****	Unlikely	MD 3.53 (-7,11 a 14.16)	168 (2)	+++Very low
Change in HbA1c	Serious* (-1)	No	No	No	Unlikely	MD −0.45 (-0.77, −0.13)	535 (5)	+ Moderate

* Most of the included studies did not report about allocation concealment, and they did not perform an intention-to-treat analysis.

** Presence of statistical heterogeneity (I^2^ > 75%).

*** 95% CI overlaps no effect but includes important benefit or important harm.

**** Optimal information size criterion was not meet. ADDQOL: Audit of Diabetes – Dependent Quality of Life. RR: Relative risk. MD: Mean difference.

+++ Low evidence : The authors are not confident in the effect estimate, and the true value may be substantially different from it.

++ Very low evidence: The authors do not have any confidence in the estimate, and it is likely that the true value is substantially different from it.

+ Moderate evidence: Further research is likely to have an important impact on our confidence in the estimate of effect and may change the estimate.

## DISCUSSION

Most individuals with DM1 have a hard time managing fasting and postprandial blood glucose levels. In addition, many patients with this disease have poor compliance to dietary advice.

Poor disease control can increase the risk of complications, such as retinopathy and other microvascular conditions (3). Ahola and cols. (32) reported that only one-third of patients maintained controlled blood glucose levels after a meal and that approximately 40% experienced frequent hyperglycemia despite having seemingly normal metabolic control. As such, the search for tools to improve these health issues has increased, and CHOC may be the only effective option for adherence to dietary requirement in patients with variable dietary habits.

To reduce postprandial blood glucose, protocols from the DAPHNE program and the Diabetes Teaching and Treatment have used the CHOC method for nutritional counseling (33). Some studies reported that CHOC can provide better glycemic control and lead to an improved QOL for patients (3,17). Patients using this method have greater flexibility in food choices without the concern of postprandial hyperglycemia given that the amount of carbohydrates ingested is considered when computing the amount of insulin to be administered before meals.

In daily clinical practice, the goal is to maintain good long-term disease control, prevent chronic complications from DM, and reduce the frequency of hypoglycemia to improve overall QOL. We performed a systematic review focusing on the efficacy and safety of the CHOC method in the management of patients with DM1. We included randomized trials that compared the CHOC method with conventional nutritional guidance in the treatment of patients with DM1.

Five studies met the established inclusion criteria and were included in qualitative and quantitative analyses. Most of the studies assessed changes in HbA1c, frequency of hypoglycemia, and QOL as primary endpoints. Meta-analysis showed a significant difference in final HbA1c favoring the intervention group.

A criticism of HbA1c is that even though levels are associated with the frequency of chronic complications and rate of morbidity and mortality, the value of this laboratory outcome is often discussed without considering glycemic variability. Although it is important for HbA1c levels to be lower than the cutoff values that indicate disease control, blood glucose levels can range from high to low. An association between glycemic variability and development of micro diabetes-related complications has been shown in type 2 DM and has also been studied as a possibility in DM1 (34). If confirmed, HbA1c values in DM1 would be inadequate to determine the superiority of one treatment to another. However, in this present review, the frequency of hypoglycemia was the same between the groups, which means that the relevance of lowering HbA1c would not be reduced.

Regarding QOL outcomes, several different instruments were used in the studies included, which negatively affected the single meta-analysis of this parameter. However, independent of the instrument used, an improvement in QOL from baseline compared with the final visit in most of the studies was noted, although no difference was observed between the intervention and control groups. Improvement in QOL can be more associated with follow-up programs and nutritional guidance than the initial methods evaluated.

Applying the GRADE approach for the outcomes “change in HbA1c” and “severe hypoglycemia,” it was necessary to rate down for the risk of bias because five out of the six studies lost patients to follow-up without an intention to treat analysis. In addition, concealment allocation and randomization processes were unclear in two of the studies. Similarly, imprecision was rated down for both, because optimal information size criterion was not met and 95% CI overlaps no effect but includes important benefit or important harm, respectively. Rating down for indirectness and publication bias was unnecessary. The quality of evidence for “change in HbA1c” and “severe hypoglycemia” was moderate and low, respectively, indicating that further research is likely to have an important impact on our confidence and authors are not confident in the effect estimate. The quality of the evidence regarding QOL outcomes was very low, and any estimate of its effect is uncertain.

Bell and cols. (35) recently published a similar systematic review. They included a study that was not included in our analysis because the comparison groups were different from the proposed PICO (31) They also included another study that we excluded because patients in the control group were predominantly children, and they were provided nutritional guidance of low glycemic index (36). Finally, Bell and cols. did not use the GRADE. The results of the previous study favored the intervention group, and the authors interpreted the results in support of recommending CHOC instead of general dietary advice in patients with DM1.

Considering the studies included in the present systematic review, the meta-analysis showed evidence favoring the use of CHOC in the management of adult patients with DM1. However, this benefit was limited to final HbA1c, which was significantly lower in the CHOC group than in the control group. Therefore, new randomized trials with greater internal and external validation and long-term outcomes are needed to analyze whether or not a significant difference exists between these two nutritional guidance tools in terms of other important diabetes-related outcomes, such as mortality, QOL, and diabetes complications.
